# The pathway to health in all policies through intersectoral collaboration on the health workforce: a scoping review

**DOI:** 10.1093/heapol/czae046

**Published:** 2024-11-16

**Authors:** Tara Tancred, Margaret Caffrey, Michelle Falkenbach, Joanna Raven

**Affiliations:** Department of International Public Health, Liverpool School of Tropical Medicine, Pembroke Pl, Liverpool L3 5QA, United Kingdom; Department of International Public Health, Liverpool School of Tropical Medicine, Pembroke Pl, Liverpool L3 5QA, United Kingdom; European Observatory on Health Systems and Policies, Eurostation (Office 07C020), Place Victor Horta/Victor Hortaplein, 40 bte 30 1060, Brussels, Belgium; Department of Health Management and Policy, University of Michigan, School of Public Health, 1415 Washington Heights, Ann Arbor, MI 48109, United States; Department of International Public Health, Liverpool School of Tropical Medicine, Pembroke Pl, Liverpool L3 5QA, United Kingdom

**Keywords:** Health workforce, health in all policies, intersectoral, multisectoral, governance, scoping review, global health

## Abstract

The health workforce (HWF) is a critical component of the health sector. Intersectoral/multisectoral collaboration and action is foundational to strengthening the HWF, enabling responsiveness to dynamic population health demands and supporting broader goals around social and economic development—such development underpins the need for health in all policies (HiAP). To identify what can be learned from intersectoral/multisectoral activity for HWF strengthening to advance HiAP, we carried out a scoping review. Our review included both peer-reviewed and grey literature. Search terms encompassed terminology for the HWF, intersectoral/multisectoral activities and governance or management. We carried out a framework analysis, extracting data around different aspects of HiAP implementation. With the aim of supporting action to advance HiAP, our analysis identified core recommendations for intersectoral/multisectoral collaboration for the HWF, organized as a ‘pathway to HiAP’. We identified 93 documents—67 (72%) were journal articles and 26 (28%) were grey literature. Documents reflected a wide range of country and regional settings. The majority (80, 86%) were published within the past 10 years, reflecting a growing trend in publications on the topic of intersectoral/multisectoral activity for the HWF. From our review and analysis, we identified five areas in the ‘pathway to HiAP’: ensure robust coordination and leadership; strengthen governance and policy-making and implementation capacities; develop intersectoral/multisectoral strategies; build intersectoral/multisectoral information systems and identify transparent, resources financing and investment opportunities. Each has key practical and policy implications. Although we introduce a ‘pathway’, the relationship between the areas is not linear, rather, they both influence and are influenced by one another, reflecting their shared importance. Underscoring this ‘pathway’ is the shared recognition of the importance of intersectoral/multisectoral activity, shared vision and political will. Advancing health ‘for’ all policies—generating evidence about best practices to identify and maximize co-benefits across sectors—is a next milestone.

Key Messages

- Intersectoral/multisectoral collaboration and action is foundational to strengthening the health workforce (HWF), and a strong HWF is integral to achieving health in all policies (HiAP).
- Much can be learned from examples of intersectoral/multisectoral collaboration and activity for the HWF about how to advance HiAP.
- The ‘pathway to health in all policies’ can be achieved through sound coordination and leadership, strengthened capacities, intersectoral/multisectoral strategies, information systems that span different sectors, and adequate financing and investment.
- Underscoring this pathway is the importance of political will, shared vision and the realization of co-benefits to all contributing sectors

## Introduction

Health in all policies (HiAP) is defined as follows:

*… an approach to public policies across sectors that systematically takes into account the health implications of decisions, seeks synergies, and avoids harmful health impacts in order to improve population health and health equity. As a concept, it reflects the principles of: legitimacy, accountability, transparency and access to information, participation, sustainability, and collaboration across sectors and levels of government*.(World Health Organization, 2014, p. 7)

At the heart of HiAP is intersectoral and multisectoral collaboration and action, recognizing that the health sector both influences and is influenced by other sectors. A key aspect of the health sector is the health workforce (HWF).

A holistic response to the ever-changing health dynamics within any population requires, among other factors, a strong HWF. Although the ‘health and care’ workforce also includes informal carers, who play a critical role in supporting care at home and minimizing strain on the health system, this group is largely excluded from the literature. As such, the HWF is understood here as all workers within health systems—including employed, informal and community health workers—however, with a focus on physicians and allied clinical cadres, nurses and midwives, dentists and pharmacists, around which much literature is centred (George *et al*., 2017). The new European Union Global Health Strategy, for example, launched in November 2022, promotes HiAP. It identifies a skilled labour force as one of the three key enablers for better health (European Commission, 2022).

Broader goals around primary health care (World Health Organization & UNICEF, 2018) and universal health coverage (UHC2030, 2021), which are instrumental to supporting population health, can only be achieved with an adequately capacitated and resourced HWF (Cometto *et al*., 2020; Foreign Commonwealth & Development Office, 2021; UHC2030, 2021). Research has identified a shortfall of 6.4 million physicians, 30.6 million nurses and midwives, 3.3 million dentistry professionals and 2.9 million pharmaceutical professionals that are needed in order to support realization of at least 80/100 on a universal health coverage effective coverage index globally (Haakenstad *et al*., 2022). Coronavirus disease 2019 (COVID-19) exacerbated many shortcomings in the HWF and highlighted the need for adaptable and resilient health systems (Nagesh and Chakraborty, 2020; Kuhlmann *et al*., 2021). The need for recruitment, retention, reactivation and investment strategies for a strong HWF is clear.

**Figure 1.**
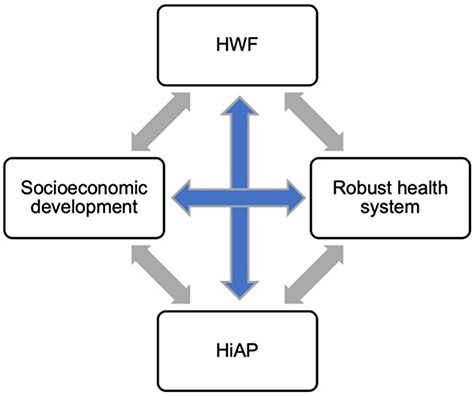
The relationship between the HWF, health systems, HiAP, and social and economic development

A well-functioning HWF that can support the aim of improving population health will also be necessary to fulfil broader aims around social development as outlined in the Sustainable Development Goals, which are central to HiAP (Greer *et al*., 2022). As shown in Figure 1, there is a relationship between the HWF, the health system, HiAP and social and economic development. The HWF is essential for health systems functioning, and a well-functioning health system better supports and retains the HWF. Investments in health are reported to yield a nine-to-one return. For example, across low- and middle-income countries (LMICs) in the early 2000s, approximately a quarter of economic growth was linked to improvements in health (World Health Organization, 2016b). A strong HWF with good retention of employees will minimize lost investments in education and professional development due to out-migration—a fully employed HWF contributes significantly to social and economic benefits of their communities and countries (The Health Foundation, 2019). For example, the National Health Service in the UK is the country’s biggest employer, with 1.6 million employees, many of whom represent demographic minorities such as women and migrants—in addition to the provision of health services, their tax and other contributions to the UK are significant (Office for National Statistics, 2023). Socio-economic development, barring some exceptions, generally liberates resources and translates into gains for the health system (Papanicolas and Smith, 2013). A robust health system is necessary to respond to population-level health needs, but to do so requires HiAP (McQueen *et al*., 2012). HiAP further supports both a resilient and sufficiently staffed HWF, alongside the achievement of broader social and economic development goals (The Health Foundation, 2019).

It has been increasingly recognized that, for the HWF to be strengthened, the health sector must work collaboratively with other sectors and stakeholders—including other government sectors, professional associations, education and training institutions, non-governmental organizations, the private sector, labour unions, community/citizen organizations, bilateral and multilateral development partners and international organizations (World Health Organization, 2016a; 2018a). To support the HWF, there is a need to decompartmentalize HWF governance across sectors to avoid siloed decision-making and strategies that are not coherent or congruent with overarching policy aims and to adopt a whole-of-government approach (Lim and Lin, 2021). Furthermore, policy coherence, as reflected in the creation of mutually reinforcing policies across government departments—and sectors—that create synergies that support HWF strengthening, while minimizing potentially negative knock-on effects, is of critical importance (Organization for Economic Co-operation and Development, 2016).

Much of the evidence around HiAP has a strong high-income country focus. For example, three recent scoping reviews of HiAP in practice had either no or exceedingly minimal LMIC representation (Guglielmin *et al*., 2018; Van Vliet-Brown *et al*., 2018; Lilly *et al*., 2023). Although there are many calls for HiAP recognizing its conceptual importance, there are fewer examples of how to move towards HiAP practically.

The ‘health’ in HiAP refers to any aspect of the health sector. Here we synthesize evidence around intersectoral/multisectoral collaboration for the HWF, as a key aspect of the health sector (World Health Organization, 2010). As outlined, the HWF is a useful exemplar, as it has unique complexities around necessary collaboration across sectors, but also has implications as a health sector component influencing wider socio-economic development, which is at the heart of HiAP. With the aim of supporting action to advance HiAP, here we present findings from a scoping review, underscoring what has worked, across different contexts, globally, to progress intersectoral/multisectoral collaboration for the HWF, framed as a ‘pathway to HiAP’.

## Methods

### Study design

Scoping reviews are useful when scoping a wide body of literature to clarify concepts and identify gaps (Munn *et al*., 2018). We carried out a scoping review with three complementary steps to identify both journal articles and grey literature, with a focus on elements of intersectoral and multisectoral activities for the HWF. Our overarching question was what can we learn from intersectoral/multisectoral activities for the HWF? We deemed a systematic review to be inappropriate due to the openness of our research question and our expectation that we would draw from a wide body of highly varied documents and study types.

Here, we understand ‘intersectoral/multisectoral’ as encompassing different government sectors, but also non-governmental organizations, professional associations and labour unions, the private sector, community/civil society organizations, bilateral and multilateral development partners and international organizations. We refer to both intersectoral and multisectoral, as much literature encompasses both or uses the terms interchangeably. There is also not necessarily total agreement about the definitions of these terms, with some authors viewing them as ‘health-led’ (intersectoral) and ‘non-health-led’ (multisectoral), intersectoral as a subset of multisectoral, or intersectoral being intentional and coordinated activities between public and/or non-public sectors and multisectoral involving those approaches between sectors that are less deliberate (de Leeuw, 2022).

### Search strategy

We followed three steps. In the first step, we built from a previous document review carried out by Martineau et al (2022) and members of the study team (MC, JR) about intersectoral mechanisms for HWF governance, which identified both journal articles and grey literature. In this document review, publications and reports were found by searching PubMed/MEDLINE using the following string searches in Table 1 and Google Scholar. In Google Scholar, Boolean operators were applied, using OR (as in Table 1) between search terms within square brackets for the key concepts of collaboration, department, governance and the HWF. These key concepts were then connected using OR or AND. For example, [coordination OR collaboration OR partnership OR stakeholder OR committee OR ‘technical working group’] OR [unit OR department OR section OR division OR office] OR [governance OR management] AND [‘human resources for health’ OR ‘health workforce’ or ‘health personnel’ or ‘health staffing’].

**Table 1.** String searches for journal articles

1	coordination OR collaboration OR partnership OR stakeholder* OR committee OR ‘technical working group’
2	unit OR department OR section OR division OR office
3	governance OR management
4	(#1) OR (#2) OR (#3)
5	(#4) AND (‘human resources for health’ OR ‘health workforce’ OR ‘health personnel’ OR ‘health staffing’)
6	From 2004–2021

Grey literature was found by carrying out site-specific searches within the domains listed below using slightly less complex terms broken up into three complementary searches for each domain due to the reduced capacity of the search functions in Google. These searches were the following (using the who.int domain as an example):

- site:who.int [coordination OR collaboration OR partnership OR stakeholders OR committees] AND [‘human resources for health’ OR ‘health workforce’ OR ‘health personnel’ OR ‘health staffing’],
- site:who.int [unit OR department OR section OR division OR office] AND [‘human resources for health’ OR ‘health workforce’ OR ‘health personnel’ OR ‘health staffing’] and
- site:who.int [governance OR management] AND [‘human resources for health’ OR ‘health workforce’ OR ‘health personnel’ OR ‘health staffing’].

Website domains of the following were searched:

- WHO headquarters and regional offices,
- Global Health Workforce Network (and its predecessor the Global Health Workforce Alliance),
- The Asia Pacific Action Alliance on Human Resources for Health
- Contemporary global HRH projects: CapacityPlus and HRH2030 and
- The World Bank.

All searches were restricted to a custom range from 2004 to 2021 and results were sorted by relevance. All of the returned documents were reviewed until it was clear that they were of no further relevance.

Across PubMed/MEDLINE, Google Scholar and site-specific searches in the original document review, the following inclusion and exclusion criteria were applied.

#### Inclusion criteria

- Context: governance of human resources for health through stakeholder coordination and management or using HR units,
- primary research studies of any design, qualitative or quantitative,
- commentaries, reports or other studies that did report on primary data/review existing data,
- articles in English language,
- articles with available full texts and
- published articles or documents since 2004.

#### Exclusion criteria

- Not related to human resources for health,
- not looking at stakeholder coordination or management, including using HR units,
- before 2004 and
- not in English.

We then screened the 95 publications from this prior document review against our present review’s inclusion and exclusion criteria described below and included 53 publications.

In the second step, we included further key HWF grey literature and journal articles that had been published since the first document review (post-June 2021), generated through discussion with subject experts from the European Observatory on Health Systems and Policies and the World Health Organization. We reviewed these against the inclusion criteria and included all 22 suggested documents.

In the third step, as gaps in the literature emerged, we carried out targeted searches, using key terms in both PubMed/MEDLINE and Google Scholar. For example:

- [investment OR cooperation OR ‘co-funding’ OR collaboration] AND [‘cross-sectoral’ OR multisectoral OR intersectoral] AND [‘health and care workforce’ OR ‘health workforce’ OR ‘healthcare workers’ OR ‘health workers’].

We identified 508 documents, screened them against the inclusion criteria and included 18 documents.

#### Inclusion and exclusion criteria

Across all three steps, we screened documents using the following inclusion criteria. These were applied on the title of the document and to abstracts (for journal publications) and executive summaries (for reports and other grey literature), and then again on full texts of any documents passing this initial screening:

- must be in English,
- must focus on intersectoral/multisectoral collaboration/engagement/activity for the HWF,
- must reflect planning, development, supporting or sustaining the HWF,
- must involve intersectoral/multisectoral activities for education, employment, retention or sustainability of the HWF and
- must be published between 2013 and 2023.

We excluded documents that did not mention the HWF or focused only on informal care providers that do not fall within the remit of the health sector. We excluded documents such as abstracts or conference proceedings that did not contain sufficient information around the context, time frame and the type of health workers involved. Searches across the different steps had considerable duplication, which was the other reason for exclusion.

### Data extraction, analysis and synthesis

We then carried out a framework analysis, adopting an analytical framework a priori. No suitable pre-existing frameworks existed. As such, our analytical framework was adapted from the World Health Organization (2018b). We selected this document due to its emphasis on ‘implementation’, as we wanted to generate evidence that was not conceptual, but practical. In this document, clear recommendations emerged around the importance of key areas to advance HiAP; these became the overarching categories around which our data were extracted and analysed: commitment and leadership, integrating intersectoral action, intersectoral governance, resource mobilization and investment, using evidence and information, sustaining activity and supporting policy coherence.

We thoroughly read and re-read the selected documents. We extracted data relevant to each overarching category in the framework that emphasized examples of practices that have been adopted, or lessons that have been learned from undertaking specific action (referred to as ‘charting the data’). We then coded extracted data and synthesized it to generate cross-cutting themes that resonated across categories, which were agreed as a study team. These themes were phrased as recommendations. Five clear recommendations emerged from our review to support the implementation of HiAP: ensure robust coordination and leadership; strengthen governance, policy-making and implementation capacities; develop intersectoral/multisectoral strategies; build intersectoral/multisectoral information systems and identify transparent, resourceful financing and investment opportunities. We have organized these findings as a ‘pathway to HiAP’, recognizing that they are all essential in the achievement of HiAP.

This work was exempt from institutional ethical review, as it engaged only with secondary data available in the public domain.

Please see the supplementary file for the PRISMA checklist for this review.

## Results

### Document characteristics

**Figure 2.**
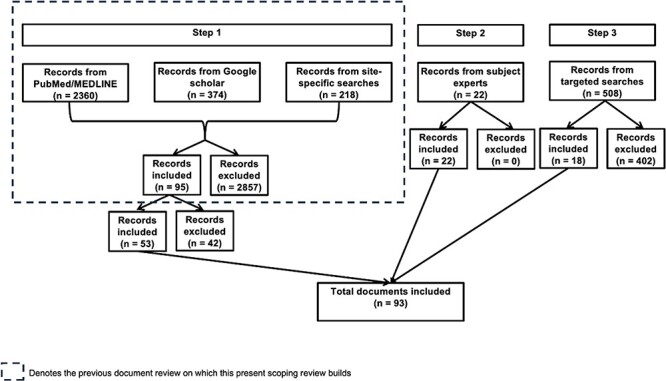
Included and excluded documents from each step of the search strategy

We included 93 documents in the scoping review (Figure 2), of which 67 (72%) were journal articles and 26 (28%) were grey literature. Most (53, 57%) came from the initial document review on which this scoping review built, with 22 journal articles and 18 grey literature documents being added through expert recommendation and additional targeted searches.

Eighteen were published from 2008 to 2013, 37 from 2014 to 2019 and 37 from 2020 onwards, reflecting an increasing trend in the amount of published relevant literature encompassing intersectoral/multisectoral activities for the HWF. One document (Global Health Workforce Alliance, n.d.) had no date. The characteristics of the documents included are found in Table 2.

**Table 2.** Document characteristics

Authors	Title	Year of publication	Type of publication	Country(ies) or regions of focus
Abuagla and Badr	Challenges to implementation of the WHO Global Code of Practice on International Recruitment of Health Personnel: the case of Sudan	2016	Journal article	Sudan
Adeloye *et al*.	Health workforce and governance: the crisis in Nigeria	2017	Journal article	Nigeria
Alonso-Garbayo *et al*.	Decision space for health workforce management in decentralized settings: a case study in Uganda	2017	Journal article	Uganda
Ayanore *et al*.	Towards resilient health systems in sub-Saharan Africa: a systematic review of the English language literature on health workforce, surveillance, and health governance issues for health systems strengthening	2019	Journal article	Sub-Saharan Africa
Badr *et al*.	Strengthening human resources for health through information, coordination and accountability mechanisms: the case of the Sudan	2013	Journal article	Sudan
Bailey and Dal Poz	Building the public health workforce to achieve health-related development goals: moving forward in collaboration	2010	Journal article	Sub-Saharan Africa
Barbazza *et al*.	Health workforce governance: Processes, tools and actors towards a competent workforce for integrated health services delivery	2015	Journal article	Europe
Baum *et al*.	Creating political will for action on health equity: Practical lessons for public health policy actors.	2022	Journal article	Global
Best *et al*.	Networks as systems: a case study of the World Health Organisation’s Global Health Workforce Alliance	2018	Journal article	Global
Bolan *et al*.	Human resources for health-related challenges to ensuring quality newborn care in low- and middle-income countries: a scoping review	2021	Journal article	LMICs
Bourgeault	A path to improved health workforce planning, policy & management in Canada: the critical coordinating and convening roles for the federal government to play in addressing 8% of its GDP	2021	Journal article	Canada
Bowser *et al*.	Global Fund investments in human resources for health: innovation and missed opportunities for health systems strengthening	2014	Journal article	Bangladesh, Ethiopia, Honduras, Indonesia, Malawi and Ukraine
Caffrey, Martineau, Ghana Ministry of Health & Ghana Health Service	Design and delivery of health workforce leadership and management professional development course for HR practitioners in Ghana	2022	Grey literature	Ghana
Cometto, Buchan, and Dussault	Developing the health workforce for universal health coverage	2020	Journal article	Global
Cometto *et al*.	Analysing public sector institutional capacity for health workforce governance in the South-East Asia region of WHO	2019	Journal article	South-East Asia
Dieleman and Hilhorst	Governance and human resources for health	2011	Journal article	Global
Dieleman, Shaw, and Zwanikken	Improving the implementation of health workforce policies through governance: a review of case studies	2011	Journal article	LMICs
Dodd *et al*.	Paris on the Mekong: using the aid effectiveness agenda to support human resources for health in the Lao People’s Democratic Republic	2009	Journal article	Laos
Dubois and Singh	From staff-mix to skill-mix and beyond: towards a systemic approach to health workforce management	2009	Journal article	Global
Effa *et al*.	Human resources for health governance and leadership strategies for improving health outcomes in low- and middle-income countries: a narrative review	2021	Journal article	LMICs
European Commission	EU Global Health Strategy: better health for all in a changing world	2022	Grey literature	Europe, global
Farrenkopf and Lee	Mapping health workforce development strategies across key global health agencies: an assessment of objectives and key interventions.	2019	Journal article	Global
Fieno *et al*.	A political economy analysis of human resources for health (HRH) in Africa	2016	Journal article	Sub-Saharan Africa
Foreign, Commonwealth & Development Office	Health systems strengthening for global health security and universal health coverage	2021	Grey literature	Global
Frenk *et al*.	Challenges and opportunities for educating health professionals after the COVID-19 pandemic	2022	Journal article	Global
Garg *et al*.	Implementing a health labour market analysis to address health workforce gaps in a rural region of India	2022	Journal article	India
Gedik and Dal Poz	Human resources for health observatories: contributing to evidence-based policy decisions.	2012	Journal article	Global
Global Health Workforce Alliance	Country coordination and facilitation: principles and process	n.d.	Grey literature	Global
Godue *et al*.	Capacity building in human resources for health: the experience of the region of the Americas	2016	Journal article	The Americas
Gopinathan *et al*.	Implementing large-scale programmes to optimise the health workforce in low- and middle-income settings: a multicountry case study synthesis	2014	Journal article	LMICs
Government of Ireland	Sláintecare: right care. Right place. Right time	2021	Grey literature	Ireland
Green *et al*.	‘Health in All Policies’—a key driver for health and well-being in a post-COVID-19 pandemic world	2021	Journal article	Global
Greer *et al*.	From health in all policies to health for all policies	2022	Journal article	Global
Gupta *et al*.	Human resources for health in major national policies and plans of Nepal	2013	Journal article	Nepal
Hastings *et al*.	Mind the gap: governance mechanisms and health workforce outcomes	2014	Journal article	Australia, Canada, the Netherlands, New Zealand, the UK and the USA
Hazarika	Health workforce governance: key to the delivery of people-centred care	2021	Journal article	Global
Houses of the Oireachtas	Committee on the future of healthcare: Sláintecare report	2017	Grey literature	Ireland
Kanchanachitra *et al*.	Human resources for health in southeast Asia: shortages, distributional challenges, and international trade in health services	2011	Journal article	South-East Asia
Kaplan *et al*.	Human resource governance: what does governance mean for the health workforce in low- and middle-income countries?	2013	Journal article	LMICs
Kigume and Maluka	Health sector decentralisation in Tanzania: Analysis of decision space in human resources for health management	2019	Journal article	Tanzania
Kingue *et al*.	Strengthening human resources for health through multisectoral approaches and leadership: the case of Cameroon	2013	Journal article	Cameroon
Kroezen *et al*.	The Joint Action on Health Workforce Planning and Forecasting: results of a European programme to improve health workforce policies	2018	Journal article	Europe
Kuhlmann *et al*.	Why we need multi-level health workforce governance-case studies from nursing and medicine in Germany	2015	Journal article	Germany
Kuhlmann *et al*.	A call for action to establish a research agenda for building a future health workforce in Europe	2018	Journal article	Europe
Kuhlmann and Larsen	Where health workforce governance research meets health services management	2016	Journal article	Europe
Kurniati *et al*.	Strengthening Indonesia’s health workforce through partnerships	2015	Journal article	Indonesia
Lim and Lin	Governance in health workforce: how do we improve on the concept? A network-based, stakeholder-driven approach	2021	Journal article	Global
Manafi *et al*.	Assessing the governance of human resources for health in Iran: a qualitative study	2019	Journal article	Iran
Marchildon *et al*.	Canada: health system review	2020	Journal article	Canada
Martineau and Caffrey	Technical Brief 9: human resources for health (HRH) strategic planning	2008	Journal article	Global
Martineau *et al*.	Improving health workforce governance: the role of multi-stakeholder coordination mechanisms and human resources for health units in ministries of health	2022	Journal article	Malawi, Nepal and Sudan
Martineau *et al*.	Strengthening health district management competencies in Ghana, Tanzania and Uganda: lessons from using action research to improve health workforce performance	2018	Journal article	Ghana, Tanzania and Uganda
Martiniuk *et al*.	Hypothesis: improving literacy about health workforce will improve rural health workforce recruitment, retention and capability	2019	Journal article	Australia
McDaid	Joint budgeting: can it facilitate intersectoral action	2012	Journal article	Global
Mikkelsen-Lopez *et al*.	An approach to addressing governance from a health system framework perspective	2011	Journal article	LMICs
Mintrom and Norman	Policy entrepreneurship and policy change	2009	Journal article	Global
Munywoki *et al*.	Tracking health sector priority setting processes and outcomes for human resources for health, five-years after political devolution: a county-level case study in Kenya	2020	Journal article	Kenya
Naccarella *et al*.	Is health workforce planning recognising the dynamic interplay between health literacy at an individual, organisation and system level?	2015	Journal article	Australia
Nyoni and Gedik	Health workforce governance and leadership capacity in the African Region: review of human resources for health units in the ministries of health	2012	Journal article	Sub-Saharan Africa
Okech *et al*.	Human resources for health coordination mechanisms: lessons from Bauchi and Cross River states of Nigeria	2021	Journal article	Nigeria
Organization for Economic Co-operation and Development	Better policies for sustainable development 2016: a new framework for policy coherence	2016	Grey literature	Global
Osei Afriyie *et al*.	The state of strategic plans for the health workforce in Africa	2019	Journal article	Sub-Saharan Africa
Paina *et al*.	Implementing the Code of Practice on International Recruitment in Romania—exploring the current state of implementation and what Romania is doing to retain its domestic health workforce	2016	Journal article	Romania
Prajapati *et al*.	Role of civil society in human resources for health management in Nepal	2013	Journal article	Nepal
Public Health Agency of Canada	Crossing sectors: experiences in intersectoral action, public policy and health	2007	Grey literature	Canada
Qian *et al*.	Challenges for strengthening the health workforce in the Lao People’s Democratic Republic: perspectives from key stakeholders	2016	Journal article	Laos
Rees *et al*.	The implications of COVID-19 for health workforce planning and policy: the case of Peru	2021	Journal article	Peru
Regional Committee for Africa	The African regional framework for the implementation of the global strategy on human resources for health: workforce 2030	2017	Grey literature	Sub-Saharan Africa
Te *et al*.	The impact of ASEAN economic integration on health worker mobility: a scoping review of the literature	2018	Journal article	South-East Asia
The Presidency & AU COVID-19 Commission	President Ramaphosa welcomes the Serum Institute funding initiative for establishment of AU Health Workforce	2022	Grey literature	Sub-Saharan Africa
Thuku *et al*.	Coordinating health workforce management in a devolved context: lessons from Kenya	2020	Journal article	Kenya
Tsofa *et al*.	Devolution and its effects on health workforce and commodities management—early implementation experiences in Kilifi County, Kenya	2017	Journal article	Kenya
van de Pas *et al*.	Health workforce development and retention in Guinea: a policy analysis post-Ebola	2019	Journal article	Guinea
Van Ryneveld *et al*.	Looking back to look forward: a review of human resources for health governance in South Africa from 1994 to 2018	2020	Journal article	South Africa
Waithaka *et al*.	Prolonged health worker strikes in Kenya- perspectives and experiences of frontline health managers and local communities in Kilifi County	2020	Journal article	Kenya
Witter *et al*.	Evolution of policies on human resources for health: opportunities and constraints in four post-conflict and post-crisis settings	2016	Journal article	Cambodia, Sierra Leone, Uganda and Zimbabwe
World Health Organization	Global strategy on human resources for health: workforce 2030	2016	Grey literature	Global
World Health Organization	National health workforce accounts: a handbook	2017	Grey literature	Global
World Health Organization	Guideline on health policy and system support to optimize community health worker programs	2018	Grey literature	Global
World Health Organization	Health labour market analysis guidebook	2021	Grey literature	Global
World Health Organization	A review of the relevance and effectiveness of the five-year action plan for health employment and inclusive economic growth (2017-2021) and ILO-OECD-WHO Working for Health programme	2021	Grey literature	Global
World Health Organization	WHO guideline on health workforce development, attraction, recruitment and retention in rural and remote areas	2021	Grey literature	Global
World Health Organization	Global competency framework for universal health coverage	2022	Grey literature	Global
World Health Organization	Human Resources for Health leadership and management: a prototype curricula package: overview	2022	Grey literature	Global
World Health Organization	Strengthening the collection, analysis and use of health workforce data and information: a handbook	2022	Grey literature	Global
World Health Organization	A system of health accounts 2011: revised edition	2022	Grey literature	Global
World Health Organization	Working for Health Action Plan 2022‐2030	2022	Grey literature	Global
World Health Organization Regional Office for Africa	Health workforce recruitment and retention for COVID-19 emergency management	2021	Grey literature	Sub-Saharan Africa
World Health Organization Regional Office for Africa	The state of the health workforce in the WHO African Region	2021	Grey literature	Sub-Saharan Africa
World Health Organization Regional Office for Europe	Health and care workforce in Europe: time to act	2022	Grey literature	Europe
World Health Organization Regional Office for Europe, European Observatory on Health Systems & Policies, Greer *et al*.	It’s the governance, stupid! TAPIC: a governance framework to strengthen decision making and implementation	2019	Grey literature	Europe
World Health Organization Office for South-East Asia	Decade for health workforce strengthening in the South-East Asia Region 2015-2024; mid-term review of progress, 2020	2020	Grey literature	South-East Asia
Zapata *et al*.	The health workforce: central to an effective response to the COVID-19 pandemic in the European Region	2021	Journal article	Europe

**Figure 3.**
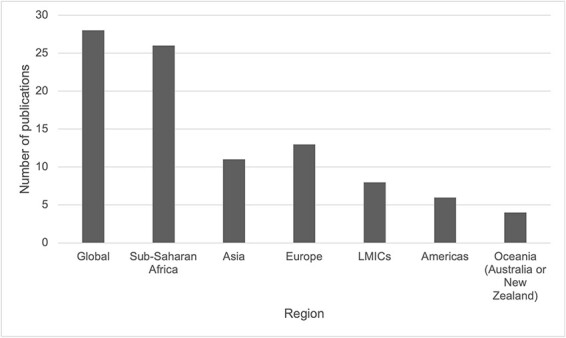
Number of publications by region

As seen in Figure 3, across documents, 28 had a global focus; 26 focused on the sub-Saharan African region or specific African countries; 13 focused on the European region or specific European countries; 11 focused on Asia (inclusive of the South-East Asian region) or specific countries from the region; eight focused on LMICs more broadly; six focused on the Americas (North and South) and four focused on Australia or New Zealand. Only one paper focused on a country from the Middle East (Iran). The total (97) is more than the overall number of papers (93), as four documents focused on more than one country or region (e.g. Hastings *et al*. (2014) included both Canada and the USA as well as Australia and New Zealand, so it is represented twice).

### The pathway to HiAP

Our analysis identified five overarching thematic areas that are essential to promoting HiAP, using the HWF as an exemplar: (1) ensure robust coordination and leadership, (2) strengthen governance and policy-making and implementation capacities, (3) develop intersectoral/multisectoral strategies, (4) build intersectoral/multisectoral information systems and (5) identify transparent, resourceful financing and investment opportunities. These are described below.

#### Ensure robust coordination and leadership

##### Uphold good governance, coordination and accountability

The importance of effective coordination and governance in intersectoral/multisectoral activities cannot be understated. The need for good governance and coordination was mentioned consistently across literature, with specific attributes being highlighted, including clarity of vision (McQueen et al., 2012; Hastings et al., 2014), coordination of intersectoral/multisectoral activities by the Ministry of Health (Barbazza et al., 2015; Hazarika, 2021) through effective stewardship—ensuring oversight, regulation of partners and deploying accountability mechanisms across partners (Hazarika, 2021); clear roles and responsibilities among all partners (Barbazza et al., 2015; Fieno et al., 2016; Lim and Lin, 2021); where useful, legal and constitutional mandates for intersectoral/multisectoral activities (Hazarika, 2021); a communication strategy enabling feedback (Hastings et al., 2014); transparency in processes (Hazarika, 2021); and monitoring and evaluation of plans and activities, and of intersectoral/multisectoral activities themselves (Dieleman and Hilhorst, 2011; Kaplan et al., 2013; Barbazza et al., 2015; Fieno et al., 2016; Cometto et al., 2019; Martineau et al., 2022b).

Common tools to support governance are strategic plans with clear indicators that can be evaluated over time, policies, operational guidelines, training manuals, codes of conduct, protocols and performance measures (Barbazza *et al*., 2015). To support monitoring and evaluation of intersectoral/multisectoral activity at the regional level, road maps and results frameworks exist for different geographical and World Health Organization (WHO) regions that can be leveraged (World Health Organization, 2022e).

An important consideration in coordination is that, in devolved/decentralized health systems, power also needs to be devolved down to subnational levels in practice such that local HWF and resource allocation decisions can be made. There needs to be greater decision-making space at these levels (Dieleman and Hilhorst, 2011; Dieleman *et al*., 2011; Badr *et al*., 2013; Munywoki *et al*., 2020; Effa *et al*., 2021). This includes state-/district-/county-level HWF plans, policies and dedicated resources (Badr *et al*., 2013). Decentralization needs to be accompanied by greater public accountability and capacity development such that more, not less, responsive service delivery can take place through decentralization (Dieleman and Hilhorst, 2011; Dieleman *et al*., 2011).

Good leadership and coordination can pay dividends around intersectoral/multisectoral collaboration. For instance, in Indonesia, government-led multi-stakeholder coordination mechanisms and processes were found to support evidence-building with intersectoral/multisectoral inputs for improved planning, implementation and monitoring of HWF programmes (Kurniati *et al*., 2015). Furthermore, joint planning was found to raise awareness among key stakeholders about the need for integrated and synergistic actions to meaningfully improve the HWF situation across the country (Kurniati *et al*., 2015).

##### Foster a shared vision

There must be a shared priority in which different stakeholders recognize the need for intersectoral/multisectoral collaboration. Following this, identifying platforms that may be capitalized on, developing shared goals and targets and developing shared coordination agreements to decrease fragmentation and duplication are important next steps for intersectoral/multisectoral decision-making (McQueen et al., 2012). Political leadership was identified by Cometto et al. as essential for mobilizing stakeholders and resources across sectors and tackling some of the rigidities in public sector regulations. In both Brazil and Thailand, specific objectives like strengthening primary care have been maintained across different political cycles through sustained intersectoral collaboration between government ministries, national agencies and local authorities (Cometto et al., 2020).

##### Engage with non-state actors

In addition to engagement with non-health sectors, there were calls across the literature for better engagement with the private sector, especially to promote a better supply of health workers (Dieleman et al., 2011; Ayanore et al., 2019; World Health Organization, 2021a; Frenk et al., 2022). While greater collaboration with the private for-profit health education sector may promote the expansion of the HWF supply, evidence suggests that inadequate regulation processes and weak capacity and governance mechanisms across government agencies and regulatory bodies lead to increased numbers of private-for-profit training institutions, but with an overall decrease in the quality of education provided (Martineau et al., 2022b). As such, intersectoral/multisectoral collaboration must also necessarily include health professional regulatory and accreditation bodies (Cometto et al., 2020).

Intersectoral/multisectoral engagement, inclusive of unions and health professional associations, is necessary to ensure that working conditions align positively with HWF expectations, which has a knock-on effect in promoting retention. For example, in Slovenia, agreements with HWF trade unions are part of the process that defines working conditions in the ‘Regulation on Continuous Health Care’ (World Health Organization Regional Office for Europe, 2022). Furthermore, addressing labour relations and promoting resolution of industrial action may also be important aspects of intersectoral/multisectoral engagement (Cometto *et al*., 2019; 2020; Waithaka *et al*., 2020).

Development partners—including donors and global health initiatives—and local and international non-governmental organizations may play an important role in health systems and HWF strengthening initiatives across settings, although their activity is particularly pronounced in fragile states, where they are key in shaping health policy. As such, they are important stakeholders to engage with in intersectoral/multisectoral activities (Witter *et al*., 2016). Mechanisms supporting exchanging information with donors that play a role in policy dialogue may support more coordinated action (Farrenkopf and Lee, 2019). The activities of large international health actors, especially donors, may not always advance intersectoral/multisectoral collaboration or health system goals. For example, overlapping or parallel streams of funding may undermine effective coordination (Farrenkopf and Lee, 2019; Cometto *et al*., 2020), lack of coordination ‘between’ different global health actors may lead to a duplication of efforts and activities (Farrenkopf and Lee, 2019), funding and support may not be coordinated with national strategic plans, causing policy incoherence and ineffective implementation (Bowser *et al*., 2014; Witter *et al*., 2016; Farrenkopf and Lee, 2019), and funding may be on annual cycles aligned with vertical programming interests, which can constrain longer-term planning and broader health systems strengthening (Dieleman and Hilhorst, 2011; World Health Organization, 2021b). This highlights the centrality of the government/Ministry of Health in coordinating multisectoral activities, including with donors. Donors, too, must embrace a shared vision.

Finally, citizen engagement during intersectoral/multisectoral activities should not be overlooked. Citizens are well placed to define the needs of communities and to provide support to the HWF. Community acceptance of health workers is important for worker motivation, performance and retention (Dieleman and Hilhorst, 2011; Prajapati *et al*., 2013; Gopinathan *et al*., 2014; Fieno *et al*., 2016; Godue *et al*., 2016; Martiniuk *et al*., 2019; World Health Organization Regional Office for Africa, 2021b; Martineau *et al*., 2022b).

##### Develop appropriate platforms to bring different stakeholders together

In order to make effective intersectoral/multisectoral decisions, the right stakeholders need to be brought together. There are a number of recognized platforms that may be leveraged to bring together different stakeholders from across relevant sectors and organizations. Committees, technical working groups, coalitions and councils with inter-ministerial or inter-departmental membership and public–private task forces are widely used (Dieleman et al., 2011; Barbazza et al., 2015). For example, in Cameroon, following years of poor stakeholder coordination, in 2010, the Ministry of Public Health mobilized a national coordinating committee for intersectoral and multi-stakeholder engagement in HWF decision-making and advocacy, which now acts as an umbrella organization that manages an HWF technical working group, a national HWF observatory and a multidisciplinary HWF research group (Kingue et al., 2013).

Health worker education and training is jointly delivered in South Africa by universities, Academic Health Complexes, nurse training institutions and the Joint Health Sciences Education Committee, which had representation from the National Department of Health and the Department of Higher Education and Training, with the National Treasury as a participating member (Van Ryneveld *et al*., 2020). This joint committee’s function is to coordinate and align policy with financing within health science education. However, this committee has not functioned optimally, due to an absence of shared vision between the sector stakeholders involved (Van Ryneveld *et al*., 2020).

In Canada, the ‘Committee on Health Workforce’ (formerly called the ‘Advisory Committee on Health Delivery and Human Resources’) was established in 2002 to establish ‘pan-Canadian’ registration and planning of the HWF. Within this committee, there are existing officials within the provincial and federal ministries of health and representatives from professional associations like the College of Family Physicians of Canada, the cross-sectoral Canadian Health Workforce Network (representing researchers, knowledge users and decision makers) and special interest organizations like the Canadian Centre on Substance Use and Addiction (Marchildon *et al*., 2020; Bourgeault, 2021).

Iceland has established a national council to provide a consultation forum to address HWF staffing and education challenges. There is wide representation across relevant sectors, including representatives from the ministries of Education and Children and Higher Education, Science and Innovation; national healthcare institutions; universities; the Icelandic Association of Local Authorities and the Directorate of Health. It aims to identify key priorities in employment and skills required, increase staffing in rural areas and carry out analytical work to support longer-term forecasting of HWF needs (World Health Organization Regional Office for Europe, 2022).

Though less common, joint ministries working towards shared goals are another platform. In Iran, which adopts a centralized model of governance, there is a joint ministry for both education and health (Manafi *et al*., 2019; Cometto *et al*., 2020). This ministry has joint responsibility for education and health through five departments (education—specifically for medical schools, research and technology, logistics, food and drugs, and health) that work together to advance national health strategic aims.

Human resources for health (HRH) units within ministries of health typically have the responsibility for core functions of HWF policy, planning and management, data management and reporting (World Health Organization, 2016a). These units then liaise with other stakeholders as needed. In Sudan, coordinated through an established HRH Unit, stakeholder forums and multisectoral planning groups collectively worked on HWF issues. The stakeholder forum convened people from different ministries, government institutions, health worker registration councils, professional associations, non-governmental organizations and some private sector institutions. There was an emphasis also on interprofessional learning and network-building. Budgets were shared, and the forums reduced duplication and fragmentation and encouraged coordinated implementation of co-developed plans (Badr *et al*., 2013; Abuagla and Badr, 2016). However, HRH units may be constrained in facilitating intersectoral/multisectoral activity. HRH units may not have adequate numbers and capacities among staff and high levels of turnover, including in leadership (Dieleman and Hilhorst, 2011; Fieno *et al*., 2016; Cometto *et al*., 2019). HRH units are sometimes hampered by the lack of a clear mandate and weak coordinating powers, in some cases due to the absence of supporting legal or constitutional mandates (Fieno *et al*., 2016).

For engaging non-state actors, meetings or workshops with citizen advisory committees or groups, or hosting ‘consensus conferences’ in which a panel of citizens questions experts or decision makers in a public forum may be effective (Barbazza *et al*., 2015).

All party or cross-party parliamentary committees are a useful approach for bringing together ministers, including from opposition parties, from across sectors around a common issue. A positive example of the utility of parliamentary committees and the importance of political consensus come from Ireland. Initiated by the Department of Health, the ‘Oireachtas Committee on the Future of Healthcare’ was developed, bringing together politicians from across the political spectrum to reach a consensus around how to achieve the shared goal of a universal single-tier health system (Sláintecare) (Houses of the Oireachtas, 2017). This committee contributed to the development of the Healthy Ireland Strategic Action Plan 2021–2025. Significant progress with the Sláintecare reform and innovation across health and social services was reported in 2021, including increased HWF recruitment across all service areas (Government of Ireland, 2021).

##### Advertise and demonstrate co-benefits

HiAP requires cross-stakeholder and sector engagement, however, towards a common goal with recognizable co-benefits to all parties. Cometto et al. highlight the need for building a ‘business case’ for HWF strengthening, particularly where domestic and external resource mobilization and investment are required (Cometto et al., 2020). In doing so, intersectoral/multisectoral collaboration and action/impact may improve. For example, in Sudan, to facilitate stakeholder buy-in, cross-institutional interests were identified, capacity-building opportunities were offered, free and timely access to HWF data were granted and stakeholders were given improved recognition and visibility. Intersectoral cooperation then resulted in the establishment of new health training institutions (nursing and midwifery training institutions increased from 18 to 55 and six medical schools were added), >10 000 new clinical positions sanctioned and enhanced remuneration for university academic staff. Ultimately, training capacity and HWF production and deployment improved, increasing HWF retention and production, which then benefitted the economy (Badr et al., 2013; Abuagla and Badr, 2016).

#### Strengthen governance, policy-making and implementation capacities

##### Strengthen technical skills

Within the health sector, the Ministry of Health plays an important role in providing leadership to ensure that HWF goals align with the broader health system and intersectoral/multisectoral goals, especially when advancing HiAP. To avoid misalignments, this role requires adequate capacity and a clear mandate to adopt participatory, intersectoral/multisectoral approaches (Hazarika, 2021).

Policy development and implementation is challenging and technical, with an added layer of complexity when this is an intersectoral/multisectoral process with many stakeholders involved, each with competing priorities. Developing a critical mass of ‘strategic thinkers’ for the HWF and HiAP at the country level can help to sustain longer-term strategic objectives (Martineau and Caffrey, 2008; World Health Organization, 2022b). To facilitate full participation in the intersectoral/multisectoral HWF and HiAP agenda, emerging across the literature was a clear set of skills and capacities that authors found to be of particular value. These were pitched not only at the individual level for those engaged in HWF policy-making and implementation, but at the institutional level (Nyoni and Gedik, 2012; Cometto *et al*., 2019), both within the health sector/Ministry of Health and across different ministries and sectors, including professional and regulatory bodies and professional associations and unions (Tsofa *et al*., 2017; Kuhlmann *et al.*, 2018 ; Thuku *et al*., 2020). These skills and capacities include managerial skills (Ayanore *et al*., 2019); data literacy and analysis skills (Ayanore *et al*., 2019; World Health Organization Regional Office for Europe, 2022); ‘HWF literacy’ to support the recognition of the centrality of the HWF in driving health and broader social development goals (Public Health Agency of Canada, 2007; Martiniuk *et al*., 2019; Hazarika, 2021; Martineau *et al*., 2022b); HWF planning expertise (World Health Organization Regional Office for Europe *et al*., 2019; The Global Health Observatory, 2022); policy formulation, implementation, and monitoring and evaluation skills (Osei Afriyie *et al*., 2019); and facilitation skills around participatory and inclusive processes (Hazarika, 2021).

It was recognized that the absence of these skills can constrain intersectoral/multisectoral activity (Osei Afriyie *et al*., 2019). For example, in Iran, within the ‘Ministry of Health and Medical Education’, sometimes, insufficient knowledge and skills among policymakers constrain the full utilization of this joint ministry (Manafi *et al*., 2019). To complement these capacities, some suggestions for specific individuals with niche expertise were recommended to be a part of intersectoral/multisectoral collaboration activities, including policy analysts, demographers, statisticians and informaticians (World Health Organization, 2016a). Where there is reliance on external technical assistance, there should be skill transfer to ensure local capacities are strengthened (Martineau and Caffrey, 2008; World Health Organization, 2022b).

Additionally, leaders need the skills to defend HWF policies and investments to get the support of other sectors, being able to highlight the importance of a robust HWF to achieve health goals like universal health coverage and other national planning and development outcomes (Public Health Agency of Canada, 2007). ‘Policy entrepreneurship’ directed towards intersectoral/multisectoral collaboration and action—that is, identifying policy opportunities to promote innovation —may also be particularly valuable within the sphere of intersectoral/multisectoral collaboration for HiAP (Mintrom and Norman, 2009; Fieno *et al*., 2016).

##### Utilize new and existing skill-strengthening platforms

To develop these capacities, a number of platforms and mechanisms have been used with success. For example, training is a widely used approach. In Ghana, the Liverpool School of Tropical Medicine (LSTM) worked with the Ministry of Health and the Ghana Health Service to adapt an LSTM-accredited course on HWF Leadership and Management to the Ghanaian context. The adapted HWF Leadership and Management professional development course has been delivered by facilitators trained and mentored by LSTM to over 60 HWF stakeholders from the Ministry of Health, the Ghana Health Service, the Christian Association of Ghana, health professional councils and teaching hospitals. The Ghana Health Service plans to continue to cascade the delivery of the course down to subregional levels (Caffrey and Martineau, 2022).

In Laos, capacity development frameworks have been developed for key sectors (transport, education and health) in relation to cross-cutting issues to help build managerial skills. Part of this was the development of, and training in, a database on human resources management by the Public Administration and Civil Service Authority, with support from the United Nations. This database was in turn adapted by the Ministry of Health and used to register all health workers in five provinces, providing information on workforce capacities and gaps to be used in planning (Dodd *et al*., 2009).

Mentorship and sharing of best practices can also build staff capacities, including to get stakeholders ‘on the same page’ (Effa *et al*., 2021)—in Kenya, there are ‘integrated HR information system champions’ who provide direct technical support to neighbouring counties (Thuku *et al*., 2020). The African Regional Framework for the Implementation of the Global Strategy on Human Resources for Health: Workforce 2030 includes the establishment of national HWF observatories as platforms to generate and share HWF data and best practices, supporting the development of HWF data literacy (Regional Committee for Africa, 2017). Furthermore, the WHO Regional Office for Europe has committed to supporting countries in assessing and developing improvement plans for HWF information systems to strengthen data collection and analysis for HWF planning, forecasting and decision-making (World Health Organization Regional Office for Europe, 2022).

#### Develop intersectoral/multisectoral strategies

##### Engage intersectoral/multisectoral stakeholders in the strategic planning process

A range of stakeholders should feed into strategic planning, though likely providing inputs at different time points, allowing space for consolidation and compromise (Martineau and Caffrey, 2008; Mikkelsen-Lopez et al., 2011). There are different platforms for stakeholder engagement and participation in strategy development. Although enacted at the district level, which may be more meaningful for decentralized/devolved health systems, one such novel example is the use of action cycles, in which stakeholders are brought together to problem-solve and action plan around key HWF management issues. This approach has been used in Ghana, Tanzania and Uganda with success, stimulating greater collaboration, trust, teamwork and resource mobilization across district health management teams. It also engendered a sense of strengthening decision-making space and empowerment, which supported efforts around decentralization (Martineau et al., 2018).

Although the Country Coordination and Facilitation (CCF) initiative is no longer in use, it effectively championed a coordination approach to bring together different stakeholders—including national governments, civil society, international agencies, finance institutions, researchers, educators and professional associations—to address key HWF challenges. Existing HWF committees or departments were central to the approach. A key aim was the development of HWF strategies (Global Health Workforce Alliance, n.d.). The adoption of the CCF approach was effective in a number of countries in engaging stakeholders in the development of HWF policies and strategic plans (Badr *et al*., 2013; Kingue *et al*., 2013; Kurniati *et al*., 2015; Thuku *et al*., 2020). For example, in Cameroon, development partners, the Cameroonian Ministry of Public Health, and key stakeholders—private sector representatives, decentralized local and regional authorities, civil society representatives, a member of the chambers of commerce, members of professional associations, trade unions, nursing and medical schools, and patient associations, as well as subject experts—jointly analysed key HWF gaps, maldistribution concerns and identified where financial resources needed to be mobilized. This resulted in the creation of the Douala Plan of Action and led to an HWF development strategy for 2011–2015, an increased number of training institutions for different healthcare cadres, greater recruitment and increased remuneration for health workers, and establishment of a retention policy for the rural HWF (Kingue *et al*., 2013). Part of the success of the CCF, at least conceptually, was the centrality of local government and that it did not seek to develop parallel systems, but to bring stakeholders together through existing platforms, where possible, with linkages to donor programmes to promote access to resources (Global Health Workforce Alliance, n.d.). However, evidence of continued CCF application or impact on HWF outcomes is limited, and in some instances, the CCF processes were seen as intruding within the remit of the national government and causing misalignment of priorities (Best *et al*., 2018).

#### Build intersectoral/multisectoral information systems

##### Integrate information systems

Data are necessary in order to support intersectoral/multisectoral planning and decision-making. Without appropriate coordination and data sharing, HWF data scattered among different actors and sectors will have limited linkages, constraining coherence in the policies that rely on such data, hindering effective HWF forecasting and planning (Cometto et al., 2019). Current systems, however, are highly constrained, with an over-reliance on paper-based, non-standardized data collection (Effa et al., 2021). There are also pronounced gaps in data from the private sector and on migration of the HWF, reflecting also the need for intersectoral/multisectoral collaboration in the generation of these data (Kanchanachitra et al., 2011; Gupta et al., 2013; Cometto et al., 2019; Osei Afriyie et al., 2019; Effa et al., 2021; World Health Organization Regional Office for Europe, 2022). Integration of different, often very siloed, sources of HWF information between sectors would be an asset. This may include, for example, national health accounts, payroll data, registries of professional councils, burden of disease assessments, data on trainees from education institutions and cost‒effectiveness analysis of various public health interventions (Cometto et al., 2019; Manafi et al., 2019; Van Ryneveld et al., 2020). ‘Whole sector planning’, also incorporating public, private, faith-based and non-governmental organization-based HWF data, should be enabled (Martineau and Caffrey, 2008; World Health Organization Regional Office for Europe, 2022).

Though specific to the HWF, the need for robust information systems has been recognized as a key objective of the Global Strategy on Human Resources for Health, as such information will also facilitate improved monitoring and accountability and impact assessment of national, regional and global strategies (World Health Organization, 2016a). Digital HWF registries and information systems are likely to be essential to this goal (Barbazza *et al*., 2015; World Health Organization, 2016a). National HWF observatories may also support intersectoral/multisectoral evidence generation and use for HWF decision-making (Gedik and Dal Poz, 2012). For instance, in Sudan, the National Health Workforce Observatory facilitated the generation of evidence for HWF decision-making through an intersectoral HWF committee under the National Council for Healthcare Coordination. These data, and subsequent decision-making using it, led to improved understanding of HWF remuneration, migration and dual practice workers (i.e. workers with both public and private practice) (Badr *et al*., 2013; Cometto *et al*., 2020; Martineau *et al*., 2022a). Similarly, such observatories in the Americas have worked together, regionally, to understand and address out-migration in the region, leading to responsive intersectoral action and mobilization of political and fiscal support (Godue *et al*., 2016; Cometto *et al*., 2020).

##### Standardize data collection

At national and regional levels, there may also be value in having standardized data collection. For instance, comparative regional data may be of use in pushing quality standards (Kanchanachitra et al., 2011; Cometto et al., 2020). The WHO has a number of specific initiatives that support data standardization for the HWF. These include the ‘minimum data set for the HWF registry’ (World Health Organization, 2015), which has specific HWF indicators that are meant to be drawn from existing HWF information systems; the National Health Workforce Accounts, which has 10 modules covering education, employment and population health needs (World Health Organization, 2017) and—with Eurostat and the OECD—the ‘joint questionnaire on non-monetary care statistics’, which has subsections on HWF employment, education and migration (Kroezen et al., 2018). Nine countries in Latin America and the Caribbean have developed shared HWF metrics, facilitating comparisons for benchmarking and capacity strengthening. Similarly, the Member States of the WHO South-East Asian Region identified 14 standardized HWF indicators—aligned with the National Health Workforce Accounts and the Global Strategy on Human Health Resources—to measure progress (World Health Organization Regional Office for South-East Asia, 2020). The information should create a ‘learning system’ for governance with utility for both the demand and supply sides (Hazarika, 2021). To maximize utility for planning, these data need to reflect a range of specific indicators, such that progress can be measured (Dieleman and Hilhorst, 2011).

##### Draw from existing processes and databases

An additional tool is the Health Labour Market Analysis, which draws from data across sectors to better understand HWF dynamics, and has been used to better align supply and demand in the UK (Cometto et al., 2020), Ghana, India and Lebanon (World Health Organization, 2021b; World Health Organization Regional Office for Africa, 2021b; Garg et al., 2022). Furthermore, to facilitate the establishment of HWF databases, it may be useful to work with independent organizations that produce research around HWF policy, such as the WHO Collaborating Centre on Health Workforce Planning and Research, based at Dalhousie University in Halifax, Canada, Health Education England and the Healthforce Center at University of California, USA (Cometto et al., 2020). Furthermore, there are international databases that may also be leveraged, most notably the World Bank, WHO and OECD-supported National Health Workforce Accounts, which have 78 standardized HWF indicators across 10 modules (World Health Organization, 2017). Implementation of these accounts has led to a significant improvement in the availability and quality of HWF data at the global and national levels (World Health Assembly Director General, 2022; World Health Organization, 2022c; 2023).

#### Identify transparent, resourceful financing and investment opportunities

##### Uphold transparency and accountability

Underscoring any approach taken to mobilize funds that are shared across sectors is the need for transparency and accountability—this links strongly to the Ministry of Health and other sectors (e.g. finance, labour and education), as being seen as credible actors to one another. To this end, WHO-championed National Health Accounts (World Health Organization, 2022d), which enable the systematic tracking of health expenditure, may be used to increase the transparency of financial flows from sources to providers (Kaplan et al., 2013). Intersectoral/multisectoral dialogue around a shared vision may, itself, result in investment for the HWF. For example, in Liberia, the Ministry of Health established a coordinated process for HWF policy formulation, engaging different stakeholders. They arrived at a shared vision and mobilized resources across different sources to fund their joint plan (Dieleman, 2011).

##### Pool resources

Common approaches to pooling resources to contribute to shared goals may be joint budgeting (combined budgets between one or more government departments) (McDaid, 2012), intersectoral budgets and accounting or co-funding arrangements (Barbazza et al., 2015). Resources—especially those around training and education or sharing of specialist HRH—may also be pooled through intercountry collaboration in the form of bilateral or multilateral agreements. For example, in Fiji, a medical school is accessible on a cooperative basis to nationals from other Pacific Island countries as a key training resource for doctors (Cometto et al., 2020).

##### Consolidate funding streams

In contexts where there may be a significant income within the health sector from donors, it may be challenging to consolidate these and ensure funding streams are not parallel or contradictory to one another. ‘Budget support’, or giving aid directly into a sector/intersectoral budget, can enable autonomy and flexibility in how funding is used, such that it can be responsive to emerging needs, which may be beneficial (Dodd et al., 2009). Furthermore, aid harmonization may be needed. For example, in Laos, the budget is delivered through the World Bank’s Poverty Reduction Support Operation, which is financed by the World Bank, the European Commission and Japan. However, it deploys aid harmonization—that is, aligning donor policies and financing initiatives around government priorities—led by the national government. These processes were a useful mechanism for consolidating intersectoral/multisectoral approaches to HWF planning and development initiatives across different partners and donors (Dodd et al., 2009).

##### Capitalize on regional and international funding initiatives

There are other international and regional initiatives that might generate funding for intersectoral/multisectoral action. For example, the ‘Working for Health’ programme generates a multi-partner trust fund to support the development of data, intersectoral/multisectoral engagement, development of intersectoral/multisectoral strategic plans and capacity strengthening, especially for educational institutions (World Health Organization, 2021b; 2022e). This programme supported Member States of the West Africa Monetary and Economic Union and the Southern African Development Community to develop, finance and implement comprehensive, intersectoral and integrated national HWF plans. Furthermore, a Southern African Development Community HRH strategic framework 2020–2030, developed through Member State and tripartite consultation and policy dialogue under this programme, will provide a common approach for regional investment and harmonization of HWF education, employment, governance and regulation. This framework is being further developed into a costed and prioritized implementation and investment plan (World Health Organization, 2021b). Finally, the African Union Health Workforce Task Team, formed by the African Union under the leadership of President Ramaphosa of South Africa, aims to develop a comprehensive framework to build an African healthcare workforce to support economic recovery and global health security, post-COVID-19. It has mobilized funding from the Africa CDC, the Serum Institute of India and Seed Global Health (The Presidency & AU COVID-19 Commission, 2022).

## Discussion

Although we included a large number of documents in this scoping review, many pointed to the ‘need’ for intersectoral/multisectoral activity, calling for increasing intersectoral/multisectoral collaboration and a move to HiAP, conceptualizing its importance. Far fewer articles—which we have emphasized in our results—highlight concrete examples of how this has been achieved in practice. From our review, we have developed Table 3, which identifies the five overarching areas on the pathway to HiAP that cut across the dimensions of our original analytical framework on HiAP implementation: ensure robust coordination and leadership; strengthen (governance and policy-making and implementation) capacities; develop intersectoral/multisectoral strategies; build intersectoral/multisectoral information systems and identify (transparent and resourceful) financial and investment opportunities. The table shows the ‘how’—as in, how to implement the ‘what’.

**Table 3.** The ‘what’ and ‘how’ on the pathway to HiAP

Linked domain from ‘Key Learning on Health in All Policies Implementation from Around the World’	What	How
**Theme 1: ensure robust coordination and leadership**		
Commitment and leadership Intersectoral governance Integrating intersectoral action Supporting policy coherence	Uphold good governance, coordination and accountability	Foster a shared vision Establish: – Clarity of vision, goals and goals/objectives – Stewardship – Clear roles and responsibilities – Legal and constitutional mandates where helpful – Communication strategies – Transparency in processes – Monitoring and evaluation of plans and processes – Monitoring and evaluation of intersectoral/multisectoral activities – Accountability mechanisms Supported through: – Strategic plans and indicators – Policies – Guidelines – Training manuals – Codes of conduct – Shared coordinated agreement – Protocols – Performance measures
	Engage with non-state actors, including: – Private sector – Professional regulatory and accreditation bodies – Unions – Professional associations – Development partners – Civil society	Develop appropriate platforms and mechanisms to bring different stakeholders together, including: – Committees (inter-departmental, inter-sectoral, cross-party, parliamentary and citizen advisory) – Technical working groups – Coalitions – Councils – Specialized units – Joint ministries
	Advertise co-benefits	– Identify and demonstrate co-benefits to all parties – Build a ‘business case’ for intersectoral/multisectoral collaboration and action
**Theme 2: strengthen governance and policy-making/implementation capacities**		
Commitment and leadership Intersectoral governance Using evidence and information Sustaining activity Supporting policy coherence	Develop a critical mass of strategic thinkers	Strengthen and build skills in: – Management – Data literacy and analysis – Subject-specific literacy (e.g. HWF literacy) – Planning – Policy formulation, implementation, and monitoring and evaluation – Facilitation of participatory approaches Through various platforms: – Capacity development frameworks – Development of and training in the use of databases – Mentorship – National observatories
**Theme 3: develop intersectoral/multisectoral strategies**		
Commitment and leadership Integrating intersectoral action Intersectoral governance Using evidence and information	Develop, implement, and monitor and evaluate strategic plans, integrated with other strategic plans, where possible	– Establish/support Government/Ministry of Health leadership/coordination – Leverage platforms (as mentioned earlier) existing platforms, where possible, to engage stakeholders from across sectors – Identify and mobilize financing sources – Develop benchmarks and indicators to assess progress – Report/produce evidence of results/impact
**Build intersectoral/multisectoral information systems**		
Using evidence and information	Establish: – Digital data systems – Integrated and interoperable data systems – Standardized data collection – ‘Learning systems’ that can draw from both supply- and demand-side information	– Involve stakeholders from private, public, faith-based and non-governmental organization sectors to ensure inclusion of data from across these – Leverage evidence-based and tested approaches and tools that may support standardization and transparency (e.g. the Health Labour Market Analysis and Health Workforce Accounts) – Establish national observatories or similar structures/ mechanisms to promote/ facilitate evidence generation and use – Leverage existing databases
**Theme 5: identify transparent, resourceful financing and investment opportunities**		
Commitment and leadership Resource mobilization and investment	Uphold transparency and accountability	– National health accounts
	Pool resources	– Joint budgeting – Intersectoral budgets and accounts – Co-funding arrangements – Multilateral–bilateral arrangements – Identify and capitalize on regional and international funding initiatives (e.g. Working for Health)
	Consolidate (donor) funding streams	– Aid harmonization

**Figure 4.**
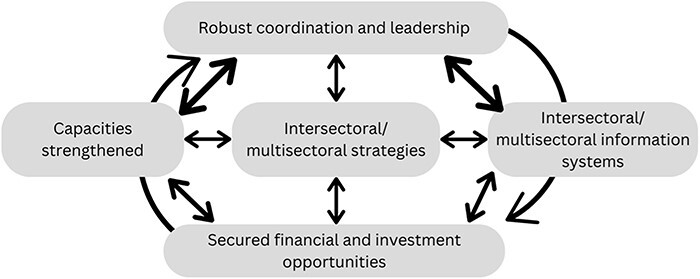
Interactions between areas on the pathway to HiAP

Although we have described this as a ‘pathway’ to HiAP, this is not linear, as these areas all interact with one another (Figure 4). For instance, it is not possible to generate a useful strategic plan in the absence of data. Nothing can be implemented without adequate funding and resource mobilization. Good governance and coordination themselves are predicated on the presence of these skills, which must be built or strengthened. As such, these areas may occur simultaneously, often reinforcing one another. In Figure 4, all of the arrows are bidirectional, highlighting the extensive interrelationships between each area.

Coming back to the domains of our original analytical framework (commitment and leadership, integrating intersectoral action, intersectoral governance, resource mobilization and investment, using evidence and information, sustaining activity and supporting policy coherence), all came out clearly from our findings, although ‘sustaining activity’ had the least explicit emphasis throughout. Though a few documents pointed at the need for a critical mass of capacitated individuals to support sustainability (Martineau and Caffrey, 2008) and risks to sustainability due to dependence on external partners—either for technical capacity or funding (Dieleman and Hilhorst, 2011), there was little written about effective designing of intersectoral/multisectoral approaches for sustainability. This reflects, more broadly, the absence of a consolidated evidence base around what works to both promote and sustain intersectoral/multisectoral collaboration for the HWF and HiAP.

Although there are other guiding documents to support the implementation of HiAP (World Health Organization, 2018b; The Health Foundation, 2019), these tend to be more high-income country focused (Gase *et al*., 2013; Rudolph *et al*., 2013; De Leeuw and Peters, 2015). Interestingly, however, although not drawing from HWF examples specifically, Gase et al. developed key recommendations for implementing HiAP that have considerable resonance with our findings: (1) develop and structure cross-sectoral relationships; (2) incorporate health into decision-making; (3) increase HWF capacity; (4) coordinate funding and investment; (5) integrate research, evaluation and data systems; (6) synchronize communication and messaging and (7) implement accountability structures. Although our findings focused less on incorporating health into decision-making—understandably, due to our explicit focus on the HWF for which this is implied—we also did not find much around synchronizing communication and messaging. Our findings instead reflect the need for making a ‘case’ or a ‘business case’, especially by identifying and communicating co-benefits across sectors.

Although it did not come out explicitly in the results as far as authors’ use of the exact terminology, a fundamental concept underlying any progress on the pathway to HiAP is political will. This has largely been reflected in our findings as fostering a shared vision. Political will is simply the commitment to achieving (policy) goals. It necessitates a shared understanding of a problem, commitment to addressing the problem and generation of a clear policy solution in response (Post *et al*., 2010). However, coming to a shared vision may be a challenge, especially in intersectoral/multisectoral collaboration, given many competing interests and priorities between stakeholders. As such, to agree to a shared vision and to galvanize political will, ‘soft skills’ in policy-making, including trust-building, communication, negotiation, consensus-building and establishment of shared values and social norms may be needed (Brinkerhoff, 2010). However, fostering political will is not restricted to the policy-making space—public interest and citizen advocacy around key issues can strengthen political will by elevating key problems or desired solutions to these into the policy-making sphere (Baum *et al*., 2022).

It is critically important to highlight that although we are describing here a pathway to HiAP, the next milestone is likely to be health ‘for’ all policies (H4AP). Although we have written about the centrality of the health sector to much intersectoral/multisectoral activity for the HWF and HiAP, many of these activities concentrate their benefits in the health sector. While their role remains fundamental, H4AP is premised wholly on bidirectional benefits, either direct or indirect (Greer *et al*., 2022; 2023). Other sectors should not only collaborate with the health sector because it benefits the health sector, but because it benefits them as well. H4AP complements HiAP by recognizing and building on co-benefits and ‘win–win’ situations. As such, growing evidence and insights around advancing mechanisms to identify and support co-benefits, which are more likely to facilitate full and sustained investments from other sectors, should be emphasized (Greer *et al*., 2023).

### Implications for practice

This scoping review has highlighted a number of practical recommendations to support intersectoral/multisectoral activity to strengthen the HWF and advance HiAP. Some core areas of focus did emerge. Capacity strengthening and development of a critical mass of ‘strategic thinkers’ with capacities in intersectoral/multisectoral working may necessitate more locally owned training opportunities. In this respect, training frameworks and mentorships may be of value (Dickin *et al*., 2019), as well as leveraging local technical experts.

Wider-scale data sharing is invaluable but is likely to be constrained by the absence of dedicated and robust digital health information systems in many settings (Kaplan *et al*., 2013). For example, a 2018 regional survey to assess the status of the HWF in the African Region found that, among the 43 countries where data were collected, 29 (67.4%) had an HWF information system or a registry with a regularly updated database; however, these were often populated through a combination of digital and paper-based registry information (World Health Organization Regional Office for Africa, 2021b). This study and others have highlighted an absence of data from the private sector (World Health Organization Regional Office for Africa, 2021b; Ahmat *et al*., 2022). The need to strengthen digital information systems is clear. To this end, though notwithstanding challenges, there are many initiatives that are driving digitization of health information (Sheikh, 2014; Mekonnen *et al*., 2022; Semyonov *et al*., 2023).

Although there is much that can be done to promote intersectoral/multisectoral collaboration for HiAP, action remains challenging (Tangcharoensathien *et al*., 2017; Synnevåg *et al*., 2018). As intersectoral/multisectoral collaboration and action may not be formalized in some health systems, existing accountability frameworks may not be amenable to cross-sector, horizontal collaboration (Public Health Agency of Canada, 2007). Furthermore, securing investment for intersectoral/multisectoral collaboration and action in perpetually resource-constrained settings with many competing priorities may be a challenge (Martineau *et al*., 2022b). However, given the centrality of the HWF to broader goals around universal health coverage, primary health care and the Sustainable Development Goals, it may be possible to leverage dedicated, but complementary, funding for these initiatives to support intersectoral/multisectoral HWF activities for the advancement of HiAP.

At the international level, there are still many settings globally that rely on donor or non-governmental organization funding to some extent (Ifeagwu *et al*., 2021; Abiddin *et al*., 2022). Funders and other global health actors may, in addition to supporting the development of HWF strategies and activities, make intersectoral/multisectoral collaboration and action—and monitoring and reporting around this—a condition of funding (Farrenkopf and Lee, 2019).

### Implications for policy

Our findings also identified a wide range of policy implications. In particular, they suggest that legal or constitutional mandates may support intersectoral/multisectoral working. Much was written about the importance of governance, coordination and accountability, especially through and by the Ministry of Health. Identifying and supporting appropriate platforms and mechanisms to engage with relevant stakeholders is key to facilitating effective intersectoral/multisectoral collaboration. There appears to be a significant role for ‘integrated’ strategic plans developed through intersectoral/multisectoral collaboration, particularly those with clear benchmarks and indicators to enable effective monitoring and evaluation. Standardized data collection at the national, regional or even international levels may be of value in promoting progress towards strategic goals. Some authors have suggested the development of global minimum HWF standards and a shared strategic framework to establish and strengthen the evidence base for responsive, sustainable and measurable HWF development (Farrenkopf and Lee, 2019). Ongoing assessment of intersectoral/multisectoral activities with a view to strengthening the most effective approaches would be of value to embed across activities.

### Areas of future research

Although many practical recommendations have been made here, a better understanding of how intersectoral/multisectoral mechanisms themselves function and what they result in would be of value. A series of systematic reviews with targeted questions that can synthesize and quantify the strength of available evidence would be helpful to this end. Determining what works and what does not, across contexts, may be helpful in better directing context-specific activities. Health policy and systems research that focuses on better understanding stakeholder engagement and complexity within intersectoral/multisectoral collaboration, underpinned by principles like systems thinking (Alliance for Health Policy and Systems Research & World Health Organization, 2009) and political economy analysis (Sparkes *et al*., 2019), would be beneficial. There is scope to innovate and share research around core areas such as capacity strengthening and generating integrated and interoperable digital information systems, which would have widespread relevance.

### Study strengths and limitations

This was a large scoping review that encompassed both journal articles and grey literature from all regions and countries of different income levels. It took a complex subject and distilled it down to key practical and policy recommendations to support HiAP. However, documents were only identified in English, constraining insights from many regions of the world—for instance, the Middle East and North Africa were very under-represented, likely in part due to exclusion of texts in Arabic. Our review searched for intersectoral/multisectoral collaboration within the HWF, rather than evidencing where HWF decision-making has been applied with a view to HiAP explicitly. However, while this may have yielded different insights, our collated findings as expressed in Table 3 have clear resonance to HiAP and intersectoral/multisectoral engagement more broadly.

## Conclusion

Intersectoral/multisectoral collaboration and action is foundational to strengthening the HWF to enable responsiveness to dynamic population health demands, and to support broader goals around social and economic development. Learning from the intersectoral/multisectoral activities to strengthen the HWF, to support HiAP, a number of key areas may be of value: ensure robust coordination and leadership; strengthen (governance and policy-making and implementation) capacities; develop intersectoral/multisectoral strategies; build intersectoral/multisectoral information systems and identify transparent, resourceful financing and investment opportunities. Although notwithstanding challenges, there are many practical options to action these overarching areas and advance the pathway to HiAP.

## Supplementary Material

czae046_Supp

## Data Availability

All data are available in the public domain.

## Abbreviations

HiAP = health in all policies

HRH = human resources for health

HWF = health workforce

LMICs = low- and middle-income countries

LSTM = Liverpool School of Tropical Medicine

WHO = World Health Organization

## Reflexivity statement

Although all authors are from European and American institutions and are all women, we come from different disciplinary backgrounds, have worked across many different contexts and are all at different levels of seniority. This manuscript and the analysis herein reflect our respective areas of interest and expertise.
